# The effects of ambient temperature and feeding regimens on cecum bacteria composition and circadian rhythm in growing rabbits

**DOI:** 10.3389/fmicb.2024.1344992

**Published:** 2024-02-27

**Authors:** Shuai He, Ke-Hao Zhang, Qiong-Yu Jin, Qiang-Jun Wang, Jie Huang, Jun-Jiao Li, Yao Guo, Peng Liu, Zhong-Ying Liu, Dan Liu, Shi-Xia Geng, Qin Li, Ming-Yong Li, Man Liu, Zhong-Hong Wu

**Affiliations:** ^1^State Key Laboratory of Animal Nutrition and Feeding, College of Animal Science and Technology, China Agricultural University, Beijing, China; ^2^College of Animal Science and Technology, Anhui Agricultural University, Hefei, China; ^3^Handan Livestock Technology Extension Station, Handan, China; ^4^National Rabbit Industry Technology System Qingdao Comprehensive Experimental Station, Qingdao, China

**Keywords:** growing rabbit, feeding regimens, cecal bacteria, seasonal variations, diarrhea

## Abstract

Seasonal environmental shifts and improper eating habits are the important causes of diarrhea in children and growing animals. Whether adjusting feeding time at varying temperatures can modify cecal bacterial structure and improve diarrhea remains unknown. Three batches growing rabbits with two groups per batch were raised under different feeding regimens (fed at daytime vs. nighttime) in spring, summer and winter separately, and contents were collected at six time points in 1 day and used 16S rRNA sequencing to investigate the effects of feeding regimens and season on the composition and circadian rhythms of cecum bacteria. Randomized forest regression screened 12 genera that were significantly associated with seasonal ambient temperature changes. Nighttime feeding reduced the abundance of the conditionally pathogenic bacteria *Desulfovibrio* and *Alistipes* in summer and *Campylobacter* in winter. And also increases the circadian rhythmic Amplicon Sequence Variants in the cecum, enhancing the rhythm of bacterial metabolic activity. This rhythmic metabolic profile of cecum bacteria may be conducive to the digestion and absorption of nutrients in the host cecum. In addition, this study has identified 9 genera that were affected by the combination of seasons and feeding time. In general, we found that seasons and feeding time and their combinations affect cecum composition and circadian rhythms, and that daytime feeding during summer and winter disrupts the balance of cecum bacteria of growing rabbits, which may adversely affect cecum health and induce diarrhea risk.

## 1 Introduction

Diarrhea is one of the most common diseases in children. Diarrhea in children seriously damages intestinal health and hinders growth and development (Mensah and Tomkins, 2003; Hanieh et al., 2021). Many epidemiological studies have shown that the occurrence of diarrhea in children and growth animals is directly related to seasonal rotations and climate change (Rowland, 1986; Windeyer et al., 2014; Uibel et al., 2022). In addition, the use of electronic products in children at night impairing sleep quality and increasing nocturnal eating behavior, thus impairing intestinal health (Afonso et al., 2022; Lepley et al., 2022). Numerous studies have ascribed the cause of seasonal diarrhea to environmental conditions influencing the variability of digestive tract bacteria, but are limited to stool samples that do not fully represent the bacterial composition of the intestinal lumen (Wen et al., 2018; Eybpoosh et al., 2021). Similarly, variations in feeding time and durations of light exposure also disrupted the rest rhythms and impacted the growth performance of developing animals (Schanbacher and Crouse, 1980; Robert et al., 1991). Disrupted rest rhythms lead to the accumulation of Reactive Oxygen Species (ROS) in the animal’s gut, causing oxidative stress and increasing the risk of death (Vaccaro et al., 2020). It remains unclear whether meal time that clash with their natural activity rhythms can disturb the balance of the cecal bacteria and heighten the risk of diarrhea, especially during periods when children and growing animals are more prone to it.

Ambient temperature possesses sufficient phenotypic plasticity mediated through gut bacteria. The same species was shaped with differential digestive characteristics at different ambient temperatures (Li et al., 2018). Cold exposure altered intestinal flora and enhanced nutrient absorption, insulin sensitivity, and white fat browning in mice (Chevalier et al., 2015). The cold environment also reshaped the gut bacteria to increase thermogenesis (Bo et al., 2019). In addition, Animals exhibit dysbiosis of the gut bacteria and remarkable changes in metabolites that damage the intestinal barrier in heat-stressed environments (Hu et al., 2022; Xia et al., 2022). Repeated hot and cold environmental temperature domestication in Mongolian gerbils alters intestinal bacteria and drives the development of host-adapted metabolic activity (Khakisahneh et al., 2020).

Light exposure has also been reported to be an upstream signal of structural variation in gut bacteria (Wu et al., 2018; Wei et al., 2020). Coordination of photoperiod with feeding time controls oscillations of gut bacteria through fluctuations in antimicrobial peptides (Talbot et al., 2020; Brooks et al., 2021; Heddes et al., 2022). Ambient light cues can also be translated into group III innate lymphocyte signaling in the gut, which plays a role in shaping and regulating the intestinal bacterial community (Godinho-Silva et al., 2019).

The suprachiasmatic nucleus (SCN) of animals in the field environment orchestrate foraging and resting behavior during the light-dark phase transition. Inverting the light and dark phases of ingestion in growing animals presents a challenge to the SCN in orchestrating whole-body rhythmic activity. The light/dark cycle regulates the rhythm of animal activity/rest by affecting the loop from the animal’s SCN to the sympathetic nerves (Scheer et al., 2005). Increased sympathetic activity in the early stages of animal wakefulness promotes enhanced thermoregulation (Guo et al., 2021). Seasonal Rotation caused by the Earth’s revolution are important regulators of the clock mechanism, integrating changes in the timing of light and ambient temperature (Arendt, 2012). Environmental temperature changes are most pronounced during the turnover of seasons, causing a shift in the thermoneutral zone and changing the metabolic intensity for the animal SCN activity of the animals (Liao et al., 2022). Daytime feeding in rodents alters the oscillatory rhythm of bile acids in the circulation (Eggink et al., 2017), which subsequently interferes with the thermogenesis and thermoregulation of brown fat G protein-coupled bile acid receptor 1 (TGR5) (Watanabe et al., 2006).

In the livestock industry, although intelligent feeding systems were introduced in the animal rearing process, the rational parameters of feeding regimens were predominantly set according to the workers’ timetable, ignoring the activity/rest phase of the animals. For rabbits in particular, daytime feeding contradicts their nocturnal activity. Daytime feeding of nocturnal animals causes bacterial imbalance and damages intestinal health (Voigt et al., 2014). The cecum bacterial community of rabbits is considered to be an important basis for maintaining digestive health and promoting animal growth (Combes et al., 2013). The symbiotic relationship between bacteria and host involves the regulation of intestinal physiological, immune and metabolic activities (Yoo et al., 2020). Numerous fecal transplantation experiments have shown that specific intestinal bacteria can improve intestinal disease and alter the metabolic profile and health of recipient animals (Allegretti et al., 2020; Craven et al., 2020; Leong et al., 2020). Factors such as food structure and environmental conditions can alter the composition and function of intestinal bacteria (Claesson et al., 2012; Deng et al., 2020). The intestinal bacterial structure of rabbits in the growing stage is exceptionally unstable, and the superimposition of weaning, cage transport and feed stress often triggers diarrhea, causing the most serious animal losses (Zhuang et al., 2022). Children, comparable to growing rabbits, are also characterized by immature gut development and gut bacterial instability (Lu et al., 2009). The disruption of intestinal bacterial homeostasis triggers the occurrence of diarrhea in a large number of children (Di Biase et al., 2021). Seasonal changes in environmental temperature and humidity are also important factors impacting the balance of the gut bacterial community in growing animals and children (Oakley et al., 2018; Chong et al., 2021). Therefore, identifying feeding regimens that enhance the stability and resilience of the gut bacteria under different seasonal environmental conditions can be an effective strategy for providing a healthy diet for children and contribute to efficient livestock production.

We have previously found that feeding growing rabbits during the day in summer can alter the cecum bacterial structure and increase the risk of diarrhea (Wang et al., 2021). In rabbit production, the use of open shed not only saves the farmer’s capital investment, but also provides sufficient sunlight and natural air for rabbits and enhances animal welfare (Rahman et al., 2018). Compared to closed rabbit sheds, rabbits survive in open sheds with greater fluctuations in light intensity, light hours and temperature, more closely resembling real seasonal environmental variations. In order to investigate the effects of ambient temperature and feeding regimens on cecum composition and circadian rhythm. This study combined 16S rRNA amplification, high-throughput sequencing, PICRUSt prediction and random forest to achieve multi-time points sample collection, analyze the differences in cecum bacterial composition and potential function of rabbits growing in open sheds, identify bacterial fluctuation characteristics associated with feeding time and look for bacteria associated with seasonal environmental temperature. Thus, this study provides a scientific basis for evaluating feeding regimens and environmental regulation in growing rabbits. This also provides important clues to assess the impact of seasonal diarrhea and disturbances in the timing of eating on cecal health in children.

## 2 Materials and methods

### 2.1 Growing rabbit feeding management

**FIGURE 1:**
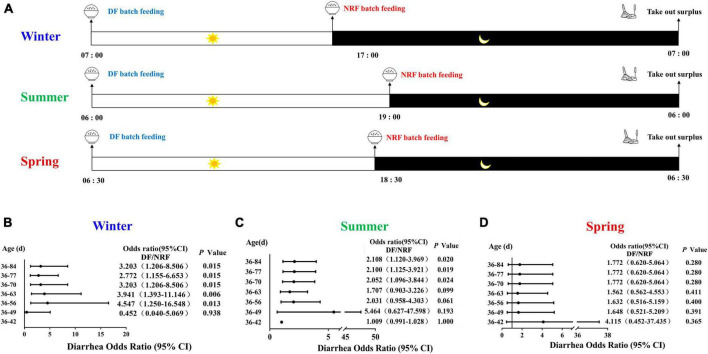
Design of animal feeding regimens and cumulative diarrhea rate of growing rabbits. **(A)** Design of rabbits feeding regimens in spring, summer and winter. Feeding time change the risk of cumulative diarrhea occurring in **(B)** winter; in **(C)** summer, results from our previous study (Wang et al., 2021); in **(D)** spring.

In the experiment, a total of 648 weaned rabbits of similar age (35 days old) and body weight (0.91 ± 0.10 kg) were selected in summer (July–August), spring (April–May), and winter (December-February) with 216 rabbits in each season. And 216 rabbits were randomly divided into two groups (daytime feeding, DF and night-restricted feeding, NRF) of 108 rabbits per group per season, and reared in an open rabbit shed with rolling plastic curtain in a commercial rabbit farm in Qingdao China. The plastic curtain was fully opened in summer. In spring and winter the plastic curtain was used to keep warm. When the temperature drops at night, the curtains were closed and were partly opened during the daytime when the temperature rises. The DF and NRF groups dropped feed before dawn and before dark, respectively, and both groups collected residual feed before dawn the following day. The DF feed delivery time was 7:00 am, 6:00 am, and 6:30 am in winter, summer and spring, respectively, and NRF feed were delivered at 5:00 pm, 7:00, pm and 6:30 pm. Both DF and NRF surplus food were collected at 7:00 am in winter, 6:00 am in summer and 6:30 am in spring (Figure 1A). The feed formulation and nutrient composition are shown in Supplementary Table 1.

### 2.2 Ambient temperature recording

The test was conducted using an automatic temperature and humidity recorder (Apresy179-TH, Apresy Precision Optoelectronics Co., Ltd., accuracy ± 0.3°C, ± 3% RH, respectively) to continuously record the temperature and humidity in the house, and the data were automatically recorded once every 10 min. In the analysis of the relationship between ambient temperature and cecum bacterial abundance, the temperature values at the moment of slaughter and at the closest distance were recorded as the room temperature at which the growing rabbits were kept.

### 2.3 Mortality and diarrhea

Death and diarrhea were recorded daily during each season of the experiment in the DF and NRF groups, respectively, cumulative mortality and diarrhea rates were calculated in 7-days increments.

### 2.4 Growth rabbit cecum contents collection

Growing rabbits were reared until 84 days of age, rabbits were euthanized by cervical dislocation at an interval of 4 h. The contents of the cecum of 36 rabbits were collected from each of the DF and NRF each season. The specific sampling operation was as follows: six collection time points (7:00, 11:00, 15:00, 19:00, 23:00, 3:00) were set to collect mid-cecum contents, six rabbits per time point as biological replicates, and stored at −80°C, for subsequent genomic DNA isolation.

### 2.5 DNA extraction and sequencing for cecum bacteria

Genomic DNA was extracted from cecum contents using the Power Soil DNA Isolation Kit (MOBIO Laboratories, Carlsbad, CA, USA) according to the manufacturer’s instructions. Forward primer 5′-ACTCCTACGGGAGGCAGCA-3′ and reverse primer 5′- GGACTACHVGGGTWTCTAAT-3′ were used to amplify the V3-V4 region of the 16SrRNA gene with a specific barcode. High-throughput sequencing was performed using Illumina HiSeq for the 2500 PE250 platform (Illumina, San Diego, CA, USA).

### 2.6 Bioinformatics analysis

Raw reads were uploaded to Quantitative Insights into Microbial Ecology (QIIME2) software, and fastq files were quality filtered, trimmed, denoised, and merged using the DADA2 package packaged with QIIME2. Clean reads were then feature-classified using DADA2 to filter out amplicon sequence variants (ASVs) with relative abundance <0.005%. The classification annotation of ASVs was based on the SILVA132 database using the naive Bayes classifier. In addition, sequence data was refined to a depth of 29675 sequences per sample to perform bacterial α and β diversity calculations. In the β diversity analysis, Principal Coordinates Analysis (PCoA) was performed based on unweighted UniFrac distances, and analysis of similarities (ANOSIM) tested for statistically significant differences between groups. In addition, we used linear discriminant analysis Linear Discriminant Analysis (LDA) to select thresholds (LDA > 3.0) and Linear discriminant analysis Effect Size (LEfSe) to select biomarkers in different groups. simca (version 13.0) was used for partial least squares discriminant analysis (Partial least squares Discriminant Analysis, PLS-DA).

Phylogenetic Investigation of Communities by Reconstruction of Unobserved States 2 [PICRUSt2 (2.3.0)] from the BMKCloud^1^ was used to predict differences in bacterial function between a single season specific time point and the remaining five time point sets.

The relative abundance of bacterial taxa in the cecum of growing rabbits at the genus level and the corresponding ambient temperature were regressed using the default parameters implemented in the R algorithm (R package randomForest ntree = 1000, using the default mtry of p/3, where p is the number of taxonomic units of the class). A random forest classifier was used to analyze the relationship between bacteria with relative abundance greater than 0.1% and temperature variation. The list of taxa ordered by feature importance from the random forest is determined over 100 iterations. The number of tagged taxa is determined by 10-fold cross-validation using the rfcv() function in the R package “randomForest,” repeated five times.

### 2.7 Jonckheere-Terpstra-Kendall cycle (JTK_Cycle) analysis

The non-parametric Jonckheere-Terpstra-Kendall cycle (JTK_Cycle) was used to analyze the significance, amplitude and phase of the 24-h cecal bacterial rhythms (Hughes et al., 2010). The relative abundance values of microorganisms sequenced at six time points during the day and night were entered into the corresponding R program of JTK_Cycle to obtain the corresponding rhythmic parameters such as period, phase and amplitude.

### 2.8 Statistical analysis

The cumulative incidence of diarrhea and death was evaluated using the ratio (OR), and the chi-square test was calculated at 95% confidence intervals (95% CI). The Wilcoxon rank sum test was used to analyze the differences in non-parametric data between the two groups. For all other data, *t*-test was used to compare the differences between the two groups, and two-way ANOVA was used to analyze the interaction between cecum bacterial composition and seasonal variation, feeding regimens, and seasonal variation and feeding regimens interactions, using SPSS 20.0 software (SPSS, Inc., Chicago, IL, USA) for statistical analysis. In addition, Spearman’s rho non-parametric correlations and *P*-values (false discovery rate corrected *P*-values) were calculated using the Psych package.^2^ Data plots were generated by Prism 7.0 software (GraphPad software, Inc., La Jolla, CA, USA).

## 3 Results

A total of 17,199,561 pairs of Reads were sequenced from 216 samples. A total of 12,628,568 Clean Reads were generated after double-ended Reads were quality controlled and spliced, generating at least 41,695 Clean Reads per sample and an average of 58,466 Clean Reads. Each cecum content sample had sufficient ASVs to reflect the maximum level of bacterial diversity, indicating sufficient sequencing depth (Supplementary Figure 1).

### 3.1 Seasonal variations induce alterations in bacterial composition

**FIGURE 2:**
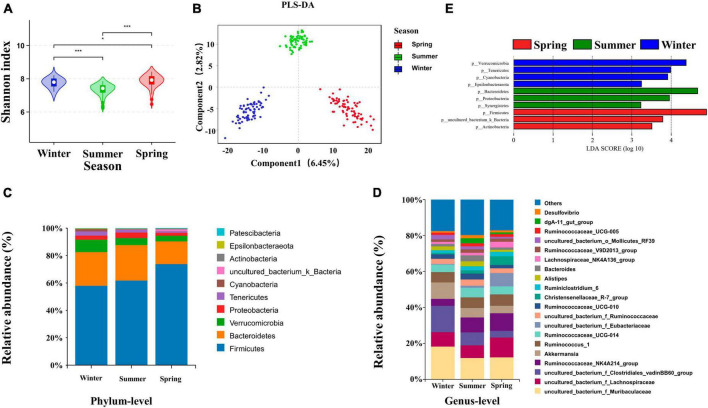
Effects of seasons on cecum bacterial structure of growing rabbits. **(A)** Shannon analysis of cecum bacteria, * represents *P* < 0.05 and *** represents *P* < 0.001. **(B)** PLS-DA analysis of the cecal bacteria. The taxonomic composition of cecum bacteria at the **(C)** phylum level (top 10, according to relative abundance) and **(D)** genus level (top 20) under different seasons. **(E)** LEfSe analysis at the genus level (LDA > 3).

Both the Shannon-Wiener diversity index (Shannon) and Abundance-based Coverage Estimator (ACE) indices were used to reflect the diversity of the cecum bacteria of rabbits grown in different seasons. Cecum bacterial α diversity was significantly higher in spring than in other seasons (*P* < 0.05) (Figure 2A). The ACE index of cecum bacteria was significantly higher in winter-grown rabbits than in other seasons (*P* < 0.05) (Supplementary Figure 2). Overall, the cecum bacterial diversity of growing rabbits was higher in spring than in other seasons, and the bacterial community richness was higher in winter than in other seasons. The weighted UniFrac was used to calculate the distance of cecum contents samples in different seasons. Analysis of Similarities (ANOSIM) analysis indicated that intergroup differences were significantly greater than the intra-group differences in different seasons. The PCoA analysis showed that the *R*-values were all greater than 0 (*P* = 0.001). PLS-DA, Non-metric Multidimensional Scaling (NMDS) and PCoA all clearly classified cecum bacteria into three different groups in different seasons, indicating significant differences in the cecum bacterial structure of growing rabbits reared in different seasons (Figure 2B and Supplementary Figures 2B, C). The top 10 dominant phyla in 84-day-old growing rabbits were composed of Phylum *Firmicutes*, *Bacteroidetes*, *Verrucomicrobia*, *Proteobacteria*, *Tenericutes*, *Cyanobacteria*, *uncultured_bacterium_k_Bacteria*, *Actinomycetes*, *Epsilonbacteraeota*, and *Patescibacteria* were the top 10 bacterial phyla in terms of abundance (Figure 2C). The overall composition was mainly composed of *Firmicutes* and *Bacteroidetes*, which together accounted for more than 80% of the bacterial composition of the rabbit cecum. In addition, the relative abundance of bacteria at the level of 20 genera was higher than 1% (Figure 2D).

To further explore the seasonal rotation of cecum bacteria composition for growing rabbits, linear discriminant analysis effect size analysis (LDA > 3) and Wilcoxon rank-sum test was performed on the bacteria in different seasons. The iconic winter genera are *Campylobacter*, *Anaerovorax*, *Butyricimonas*, *Akkermansia*, and *Roseburia*. The iconic summer genera are *dgA-11_gut_group*, *Alistipes*, *Synergistes*, *Desulfovibrio*, *Bacteroides*, *uncultured_bacterium_f_Rikenella*. The iconic spring genera are *Erysipelotrichaceae_UCG-004*, *Blautia*, and *Lachnospiraceae_NK4A136_group* (Figure 2E).

### 3.2 Dietary regimens alter the composition and circadian rhythms of cecal bacteria

**FIGURE 3:**
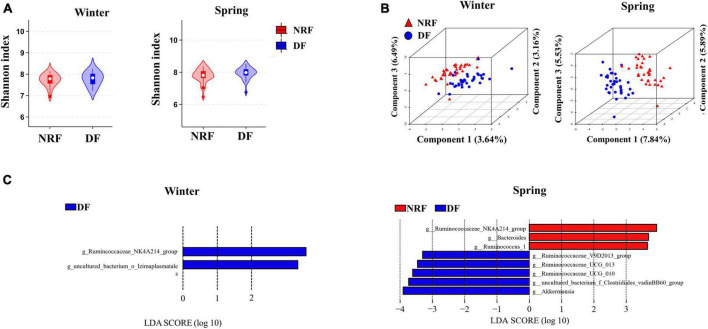
Comparison of bacterial structure under different feeding methods in winter and spring. **(A)** Shannon analysis of cecum bacteria, **(B)** PLS-DA analysis of the cecal bacteria and **(C)** LEfSe analysis at the genus level (LDA > 3).

There was no difference between DF and NRF in the comparison of bacterial α diversity either in winter or spring (Figure 3A). PLS-DA results classified NRF and DF cecum bacteria into 2 groups in winter and spring. Thus different feeding regimens altered the structure of cecum bacteria β diversity (Figure 3B). The Wilcoxon rank-sum tests were performed in winter and spring. Compared to DF, NRF significantly increased the abundance of *Lentisphaerae* phylum in winter and decreased the abundance of *Cyanobacteria* and *Patescibacteria* phylum in spring (Supplementary Figure 3). LDA effect size analysis at genus level (LDA > 3) showed that *Ruminococcaceae_NK4A214_group* and *uncultured_bacterium_o_Izimaplasmatales* were significantly enriched in DF group in winter (*P* < 0.05). In spring, *Ruminococcaceae_NK4A214_group*, *Bacteroides*, and *Ruminococcus_1* were significantly enriched in NRF group (*P* < 0.05). *Ruminococcaceae_V9D2013*, *Ruminococcaceae_UCG_2013*, *Ruminococcaceae_UCG_011*, *bacterium_f_Clostridiales_vadinBB60*, and *Akkermansia* were significantly enriched in DF group (*P* < 0.05) (Figure 3C).

**FIGURE 4:**
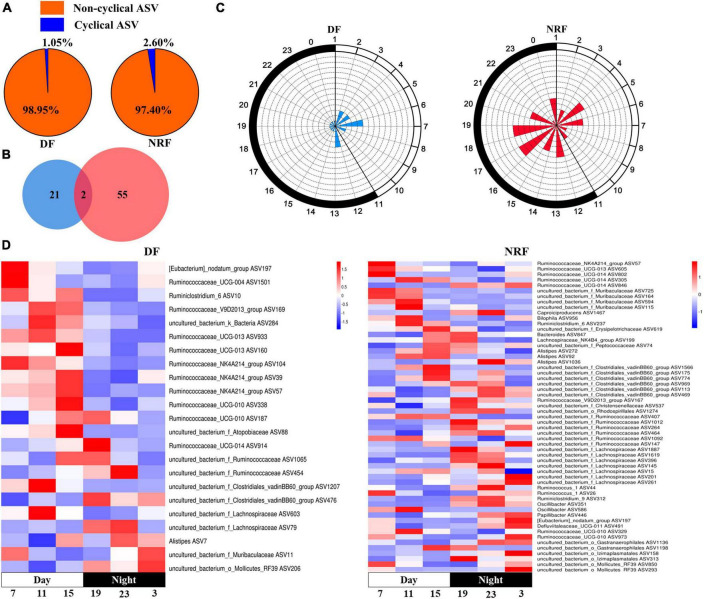
Cecal rhythmic ASVs in DF and NRF growing rabbits in winter. **(A)** Pie chart showing the proportion of rhythmic and non-rhythmic ASVs in cecum. **(B)** Venn diagram showing the number and overlapping number of rhythmic ASVs in DF group and NRF group. **(C)** A radar chart is used to display the abundance peak time of ASVs throughout the day, in which blue represents the DF group, red represents the NRF group, and the fill represents the number of rhythmic ASVs at that time point, and the time when the rhythmic ASVs peak appeared. Using JTK analysis and prediction, the radius of the dashed concentric circles represents the number of ASVs, the minimum radius unit of the dashed concentric circles represents the number of 1, and the black and white shadows on the edge of the radar map represent night and day. **(D)** Heat map showing the trend of rhythmic ASVs in growing rabbits DF group and NRF group throughout the day.

The sequenced cecal bacterial relative abundance data were analyzed by JTK to reflect the diurnal fluctuation of bacteria. In winter, 23 ASVs in DF group had circadian rhythm, accounting for 1.05% out of the total ASVs, and 57 ASVs in NRF group had circadian rhythm, accounting for 2.60% out of the total ASVs (ADJ. *P* < 0.05) (Figure 4A). Venn diagram analysis showed that most of the rhythmic ASVs were unique at different feeding regimens, and only two were rhythmic in both DF group and NRF group (Figure 4B). Radar and heat maps showed that the peak values of ASVs in DF group were mainly distributed at all-time points during the day, while those in the NRF group were mainly distributed at all-time points during the night (Figure 4C). The rhythmic ASVs of DF and NRF groups mainly belong to *Firmicutes*, *Bacteroidetes*, *Actinobacteria*, and *Proteobacteria*. Some ASVs in NRF group also came from *Actinobacteria*, *Cyanobacteria*, and *Tenericutes* (Figure 4D).

In spring, 57 ASVs in DF group had circadian rhythm, accounting for 2.60% out of the total ASVs, and 43 ASVs in NRF group had circadian rhythm, accounting for 1.96% out of the total ASVs (ADJ. *P* < 0.05). Most of the rhythmic ASVs were unique at different feeding regimens, and only five were rhythmic in both DF group and NRF group. The peak value of ASVs in the DF group was mainly distributed before and after dark, while that in the NRF group was mainly distributed before feeding at night. The rhythmic ASVs mainly belong to *Firmicutes*, *Proteobacteria*, and *Actinobacteria* in DF group. NRF group mainly belonged to *Firmicutes*, *Bacteroidetes*, *Tenericutes*, *uncultured_bacterium_k_Bacteria*, *Proteobacteria*, and *Actinobacteria* (Supplementary Figures 4A–D).

### 3.3 Interactive effects of season and feeding regimens on cecum bacterial composition

Seasonal rotation changes bacterial structure, and feeding time also changes cecum bacterial composition and diurnal oscillations. Therefore, we performed seasonal and feeding regimens interaction analyses for bacteria with relative abundance greater than 0.5% in either season (Supplementary Table 2). The results of the interaction between season and feeding regimens showed that *Ruminococcus_1* was higher in spring than in summer and winter, and NRF was higher than DF. In total, we found that bacterial abundance in nine genera was influenced by seasons, feeding patterns, and the interaction of season and feeding regimens. These included *Alistipes*, *Ruminococcaceae_UCG-013*, *Synergistes*, *[Eubacterium]_coprostanoligenes_group*, *uncultured_ bacterium_f_Clostridiales_ vadinBB60_group*, *uncultured_ bacterium_f_Rikenellaceae*, *uncultured_bacterium_o_Bacteroidales*, and *uncultured_bacterium_o_Izimaplasmatales* (Supplementary Figures 5A, B).

### 3.4 Correlation between cecum bacterial changes and diarrheal incidences

We counted the number of diarrhea occurrences throughout the feeding period and found that there was no significant difference in the risk of cumulative diarrhea between the DF and NRF groups until 49 days of age in winter growing rabbits (*P* > 0.05), and between 49 and 84 days of age, the risk of cumulative diarrhea was significantly higher in the DF group than in the NRF group (*P* < 0.05) (Figure 1B). The cumulative risk of developing diarrhea was significantly higher in the DF group of growing rabbits than in the NRF group between 63 and 84 days of age during summer growing rabbit rearing (*P* < 0.05) (Figure 1C). There was no significant difference in the cumulative risk of occurrence of diarrhea between the DF and NRF groups of growing rabbits during the entire trial period of spring growing rabbit feeding (*P* > 0.05) (Figure 1D). The comparison of total diarrhea cases and death cases in seasonal feeding practice showed that the incidence and death of diarrhea in open shed rabbits were significantly related to the season (Supplementary Table 3). The number of diarrhea in summer growing rabbits was significantly higher than in winter and spring in both the DF and NRF feed regimens (*P* < 0.001). The number of spring growing rabbits mortalities was significantly lower than in winter and summer in both the DF and NRF feed regimens. The number of mortalities was significantly lower in the winter DF group than in the summer (*P* < 0.01), while NRF reduced the number of summer mortalities resulted in no difference between winter and summer (*P* > 0.05).

We found a high tendency of diarrhea in summer. Considering that cecal bacteria are closely prioritized to cecal physiological functions, we sought to analyze the variation in cecum bacterial composition and rhythms caused by summer. We analyzed the composition at the genus level in different seasons and found that *Uncultured_bacterium_f_Barnesiellacea*, *Parabacteroides*, *Desulfovibrio*, *dgA-11_gut_group*, *Synergistes*, and *Alistipes* were significantly higher in the intestine of summer-grown rabbits compared to spring and winter (*P* < 0.05), *Escherichia-Shigella* abundance was higher in summer compared to the other two seasons. *Marvinbryantia*, *Tyzzerella_3*, and *Blautia* were significantly higher (*P* < 0.05) and *Alistipes*, *Bilophila*, and *Campylobacter* were significantly lower (*P* < 0.05) in the cecum of spring-grown rabbits than in summer and winter. Winter growth rabbits intestinal *Campylobacter*, *Akkermansia*, *Roseburia*, and *Erysipelotrichaceae_UCG-004* were significantly higher than summer and spring (Supplementary Figure 6).

Bifactorial analysis of season and feeding regimens showed that seasonal changes could significantly affect the cecum bacterial structure of growing rabbits (Supplementary Table 2). Ambient temperature was used to represent a characteristic with distinct seasonal variation. Therefore, the daily ambient temperature recorded in the rabbit shed during feeding trials completed in different seasons was used to explore seasonal bacterial variation (Supplementary Figure 7).

**FIGURE 5:**
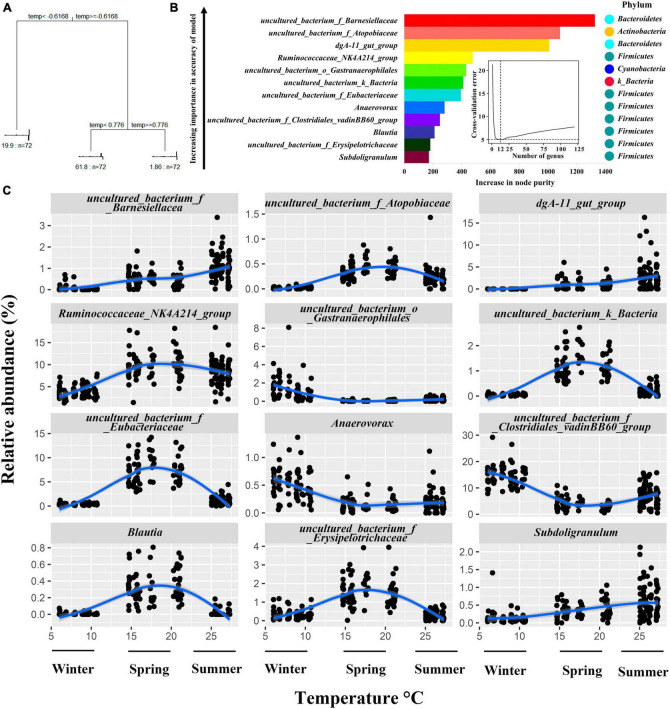
Relationship between relative abundance changes of growing rabbits cecum bacteria at the genus level and seasonal environmental temperature changes. **(A)**. MRT Analysis: correlating shed temperature with cecal bacterial genera abundance in growing rabbits. **(B)** Random Forest Analysis: assessing correlations between cecal bacteria genera variability and shed temperature in growing rabbits. **(C)** Top 12 Cecal bacteria Genera: examining temperature-dependent variations in growing rabbits’ shed environment.

The results of the Multivariate Regression Trees (MRT) analysis showed that the differences in environmental temperature in the house during the three seasons could classify the bacteria into three groups (Figure 5A). Random forest regression models of temperature and bacterial abundance across seasons explained 84.36% of the total variance, indicating a strong association between cecum bacterial variation and seasonally induced temperature changes. To reveal important bacterial taxa associated with environmental temperature changes in the house as biomarker taxa, the cross-validation error was minimized when 9 important marker taxa were used by 10-fold cross-validation, however, the number of classes against the cross-validation error curve was stable when 9–14 marker taxa were used, i.e., the desired regression results could be obtained, therefore, we selected 12 classes as the biomarker model in the taxa (Figure 5B). The top 12 bacterial taxa of the growing rabbit cecum bacteria with temperature, these genera belong to the phyla *Firmicutes*, *Bacteroidetes*, *Cyanobacteria*, *uncultured_bacterium_k_Bacteria*, *Proteobacteria*, and *Actinomycetes*. The relative abundance of cecum *uncultured_bacterium_f_Barnesiellacea*, *dgA-11_gut_group*, and *Subdoligranulum* was positively correlated with ambient temperature and was higher in the summer temperature range The relative abundance of *uncultured_bacterium_f_Atopobiaceae*, *Ruminococcaceae_NK4A214_group*, *uncultured_bacterium_k_ Bacteria*, *uncultured_bacterium_f_Eubacteriaceae*, *Blautia*, and *uncultured_bacterium_f_Erysipelotrichaceae* was higher in the spring temperature interval. The *Anaerovorax*, *uncultured_bacterium_f_Clostridiales*_*vadinBB60_group*, and *uncultured_bacterium_o_Gastranaerophilales* had higher relative abundance in the temperature interval in winter (Figure 5C).

**FIGURE 6:**
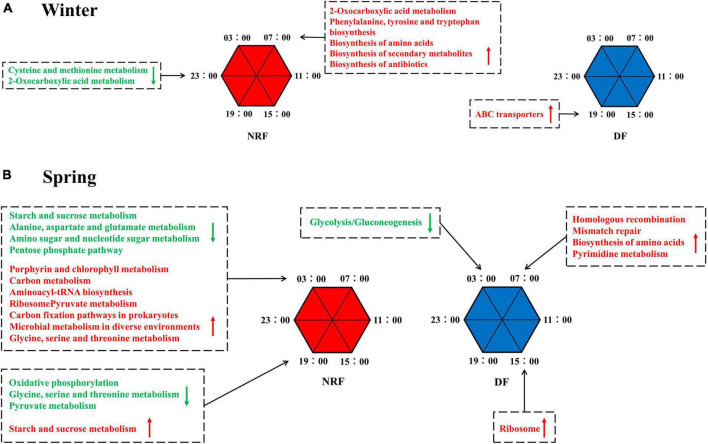
PICRUSt predicted the diurnal metabolic potential of cecum bacteria for different feeding regimens in **(A)** winter, in **(B)** spring. Green font means that the metabolic process described at that time point is weaker than the other five time points. The opposite is indicated in red font.

To explore the role of rhythmic changes in cecum bacteria, we used PICRUSt2 for functional prediction of cecum bacteria in each season. The biosynthesis potential of 2-Oxocarboxylic acid metabolism, phenylalanine, tyrosine, tryptophan, amino acid, secondary metabolite, and antibiotic biosynthesis was higher at 7:00 am than at the rest of the day during winter NRF feeding, and the intensity of cysteine and methionine metabolism, 2-Oxocarboxylic acid metabolism, was at its lowest level of the day at 11 pm. In winter DF feeding, only the ATP-binding cassette transporter (ABC transporter) was higher at 7:00 pm than at the rest of the day (Figure 6A and Supplementary Figure 8A). During spring NRF feeding, processes such as carbon metabolism and protein biosynthesis peak at 3:00 am at night. In contrast, starch and sucrose, alanine, aspartate and glutamate, amino acid and nucleotide metabolism, amino acid-tRNA biosynthesis, and the pentose phosphate pathway reach their troughs at this time of day. Starch and sucrose metabolism reached its highest value and Oxidative phosphorylation, glycine, serine and threonine metabolism, and pyruvate metabolism reached their lowest level at 7:00 pm. In the predicted model of cecum bacterial function under DF feeding in spring, gluconeogenesis/glycolysis was lower than the rest of the time at 3 am at night, and biosynthesis of amino acids reached the lowest level at 7:00 am. The peak of the day and the strongest ribosomal activity at 3:00 pm (Figure 6B and Supplementary Figure 8B).

## 4 Discussion

Daytime feeding is often used in rabbit production, which is inconsistent with their activity rhythms. We had introduced nighttime feeding in the summer to compare the differences in cecal bacterial composition with daytime feeding regimens (Wang et al., 2021). Now we also examined whether this alteration in cecal bacterial composition caused by feeding regimens was preserved in different seasonal environments.

In this study, the gut bacteria were clearly separated in the three seasons, indicating that there were significant differences in the cecum bacterial structure of growing rabbits under the three seasons. The environmental temperature is vital for the regulation of the animal’s thermal balance. In this study, the average temperature difference between summer and spring reached 5°C, and the average temperature difference between spring and winter reached 13°C. *Actinomycetes* have the function of maintaining a stable intestinal bacterial structure in response to intestinal anti-inflammation (Doaa et al., 2016), while *Proteobacteria* can result in reduced intestinal bacteria diversity and induce inflammatory processes, a phylum directly related to irritable bowel syndrome (Pittayanon et al., 2019). Therefore, the lower abundance of *Proteobacteria* and higher abundance of *Actinomycetes* observed in the spring suggest a healthier gastrointestinal environment. This supports the hypothesis that a well-balanced cecum bacteria composition is instrumental in diminishing the incidence of diarrhea and mortality. At the genus level, the relative abundance of *Blautia*, *Marvinbryantia*, and *Tyzzerella-3* was higher in spring than in the other two seasons. *Blautia* has been shown to modulate host health and attenuate metabolic syndrome (Liu et al., 2021). *Marvinbryantia* facilitates fiber breakdown, aids in the digestion of the cecum and replenishes energy (Gao et al., 2022). Related studies have reported that both *Tyzzerella-3* and *Marvinbryantia* are associated with the synthesis of SCFA (Short Chain Fatty Acids), and their elevated abundance implies an increase in the ability to maintain the intestinal barrier (Zhang et al., 2020; Gao et al., 2022). The potential pathogens *Desulfovibrio* and *Alistipes*, the diarrhea causative agent *Escherichia-Shigella* (Nataro and Kaper, 1998), were significantly higher in relative abundance in summer than in the other two seasons. *Desulfovibrio* reduces sulfate to produce H_2_S, which poisons intestinal epithelial cells and can cause intestinal sensitivity and leaky gut (Kushkevych et al., 2019). The relative abundance of intestinal *Alistipes* is significantly elevated in patients in stressful environments or suffering from chronic fatigue syndrome (Nagy-Szakal et al., 2017). The winter and summer seasons may raise stress levels due to the effects of unsuitable long-term ambient temperatures, while daytime feeding disturbs daytime rest in rabbits, causing constant stress, and may be an important contributor to elevated *Alistipes* abundance. *Alistipes* produces sulfonolipid, which induce cecal inflammation (Parker et al., 2020). Feeding time determines the circadian rhythm of the serotonin substrate tryptophan, which *Alistipes* hydrolyzes to indole, interfering with serotonin availability (Cussotto et al., 2021). Thus, *Alistipes* may reduce the availability of serotonin, upstream of melatonin synthesis, thereby reducing melatonin production and affecting gut health by influencing the intestinal clearance of ROS. However, the *dgA-11_gut_group* and *uncultured_bacterium_f_Barnesiellacea* involving in amino acid transport and metabolism, energy production and conversion (Sun et al., 2020), and protection of the intestinal barrier increases significantly during the summer. The abundance of *Bilophila*, which produces acetic, succinic, and sulfides and promotes inflammation, and *Campylobacter*, which produces endotoxins and induces severe diarrhea in animals (Rauws et al., 1988; Natividad et al., 2018). And the number of *Akkermansia*, *Roseburia*, and *Erysipelotrichaceae_UCG-004*, which have protective effects, also increased. Overall, different bacteria show differential sensitivity to such environmental changes. Growing rabbits in open shed growing rabbits have a healthier cecum bacterial community composition in spring than in winter and summer. Environmental factors during the winter and summer seasons may contribute to dysbiosis of cecum bacterial homeostasis and the increased abundance of harmful bacteria, which may be the potential cause of the increased risk of diarrhea.

And how do seasons affect the composition of cecum bacteria in growing rabbits? Of note, recent studies on giant pandas, forest and alpine musk deer point to structural and functional variation in gut bacteria due to seasonal turnover-induced changes in food types (Jiang et al., 2021; Huang et al., 2022). However, the type of feed we have supplied to growing rabbits has been consistent over the past three seasons (Supplementary Table 1). Similar to the results of a previous environmental acclimatization trial with Chinese giant salamanders, 10% of the changes in the gut bacterial community were also caused by differences in ambient temperature, even though the type of feed did not change (Zhu et al., 2021). Our study found that the abundance of cecal bacteria varies with the ambient temperature (Figure 5). It is possible that variations in ambient temperature and humidity resulting from seasonal changes may impact the growth of foodborne bacteria, despite consistent exposure durations to air (Rowland, 1986).

Temperature changes occurring as a result of seasonal rotation are an important driver of bacterial variation in the cecum of growing rabbits, after a bifactorial analysis of feeding time and seasonal differences as well as MRT analysis. The variance explained by the random forest model of ambient temperature and changes in cecum bacterial abundance of growing rabbits in each season was 84.36%, indicating a strong correlation between cecum bacterial abundance and ambient temperature changes. Season specific dominant genera such as *Blautia*, *dgA-11_gut_group*, and *uncultured_bacterium_f_Barnesiellaceae* are also marker bacteria for environmental temperature changes. Therefore, we consider changes in beneficial and harmful bacterial composition mediated by seasonal temperature changes as potential factors affecting cecum health and risk of diarrheal mortality in growing rabbits.

Regular living and rhythmic time-restricted feeding can maintain the balance of gut bacterial abundance in adult humans and animals, reducing the risk of metabolic disease and intestinal inflammation (Asher and Sassone-Corsi, 2015; Voigt et al., 2016). Our study points out that changes in feeding time affect the composition of gut bacteria. Among the top 10 phyla in terms of relative abundance in winter, the NRF group contained a significantly higher abundance of *Lentisphaerae* than that of DF. The presence of more *Lentisphaerae* in the gut of healthy subjects compared to patients with non-alcoholic fatty liver disease (NAFLD) (Jiang et al., 2015) and juvenile idiopathic arthritis (Tejesvi et al., 2016) suggests a potential protective effect of this phylum on organismal health. The *Cyanobacteria* and *Patescibacteria* phylum were significantly higher in the spring DF group. The relative abundance of these two phyla was increased in the gut of patients with ankylosing spondylitis and accompanied by a more severe organismal inflammatory signal (Liu et al., 2022). Suggesting that the DF feeding may form a potentially pro-inflammatory bacterial structure.

Gut bacterial communities follow natural circadian rhythms, and this diurnal fluctuation alters the schedule of the host’s metabolic profile (Thaiss et al., 2014). Ambient light cues can be transformed into enteric group 3 innate lymphoid cell (ILC3) signals, shaping intestinal metabolism (Godinho-Silva et al., 2019) and affecting the expression of antimicrobial peptide circadian rhythms (Brooks et al., 2021). Our results point to changes in feeding time affecting the circadian rhythm of cecum bacteria, which is consistent with previous research on feeding rhythms guiding bacterial oscillations (Thaiss et al., 2014). Summer NRF rabbits had a maximum of 4% cecal rhythm bacteria (Wang et al., 2021) and a minimum of 1.05% bacteria in DF in winter exhibited significant circadian rhythms, much lower than in adult rats (15%) and humans (10%) (Thaiss et al., 2014). The bacterial structure was further influenced by the growth phase of animals (Wang et al., 2019) and greater fluctuations in circadian ambient temperature (Grant et al., 2021). NRF raises the proportion of rhythmic bacteria in summer and winter. The results predicted by PICRUSt showed that NRF allowed peaks or troughs of bacterial metabolic activity to be enriched at nocturnal rabbit activity time phases. Similar to Palomba’s findings, changing the feeding regime affected the relative abundance and function of gut bacteria (Palomba et al., 2021). However, we dynamically recorded circadian rhythms of bacterial abundance and metabolic activity under different feeding regimes in growing rabbits. We consider that the period of the day with the highest bacterial abundance may perform more intense biological functions. Thus, NRF groups that showed more bacterial rhythmicity were enriched for richer digestive, metabolic, and immune pathways at specific time points. It may facilitate the digestion and absorption of food in animals.

The interaction of season and feeding regimens influenced the relative abundance of cecal bacteria. Seasonal and feeding time interactions were significantly associated with changes in the relative abundance of the SCFA production-associated bacterium *Ruminococcus_1*. *Ruminococcus_1* abundance of daytime feeding in spring was not significantly different among the three seasons, whereas switching to nighttime feeding significantly increased *ruminococcus_1* abundance and which was higher in spring than in winter and summer (Supplementary Figure 7). Although we have not been able to determine the exact reason for the interaction of season and feeding time on bacteria, the pairing of feeding time and different seasons predicts a diverse combination of nutritional and environmental signals. We can make two speculations. On the one hand, because of the differences in feeding time and seasons, we hypothesize that these bacteria are sensitive to both seasonal light time, light intensity, or ambient temperature, as well as to some of the nutrient signals that synchronize feeding behavior (e.g., bile acids, leptin, and insulin). And on the other hand, combinations with season and feeding time may cause nutrients favored by specific bacteria to be enriched, thus promoting bacterial proliferation.

Compared to DF, the NRF group of potentially conditionally pathogenic bacteria involving *Alistipes*, *uncultured_bacterium_f_Rikenellaceae* (Sun et al., 2017), and *Synergistes* (Borsanelli et al., 2018) showed a consistent downward trend compared to DF, while seasonal differences interfered with the magnitude of the changes. In particular, NRF decreased the most dramatically in potentially pathogenic bacteria during summer, suggesting that summer night-restricted feeding may be more beneficial for cecum health than DF.

Combined with our previous summer results (Wang et al., 2021), it was surprising that different feeding time under different seasonal environmental conditions changed the structure of the cecum microbiome community, but there was no specific pathway of bacterial community change. Alternatively, some more drastic factors during the seasonal change masked the effect of feeding time. How exactly do feeding time conditions and seasonal ambient temperatures interfere with the composition of cecum bacteria? Multiple levels of neural signaling, hormonal transmission, activity rhythms and thermoregulation may be involved in this regulatory process. Animals differ in the intensity of SCN activity in the active phase vs. the resting phase, with differences in output nerve conduction and hormone levels. Feeding time conditions that are contrary to the animal’s activity rhythms can interfere with sympathetic nerve activity and alter the circadian oscillations in body temperature (Guo et al., 2021). Long-term training in feeding patterns can stimulate the deviation of feeding phase from the active period (Chung et al., 2017). The disturbance of feeding and activity rhythm will lead to the destruction of intestinal smooth muscle contraction rhythm (Rao et al., 2001) and the accumulation of ROS in the intestinal cavity (Vaccaro et al., 2020), thus disrupting bacterial homeostasis (Ballard and Towarnicki, 2020). The secretion of melatonin is low in the day and high in the night, and the length of sunshine in different seasons also regulates the rhythm of melatonin for a long time (Adamsson et al., 2016). Studies have reported that melatonin targets enzymes that regulate bile acid synthesis (Pathak et al., 2013). The liver is infiltrated by relatively high concentrations of melatonin at night, and food entrainment placed during the day or night will alter the rhythm of bile acid synthesis and circulation patterns. Intestinal bacteria can perform bile acid biotransformation reactions, and the composition and abundance of intestinal bacteria are in turn influenced by bile acids (Cai et al., 2022). The results of the random forest reveal that 10/12 of the genera that vary with seasonal environmental temperature are from the phyla *Bacteroidetes*, *Firmicutes*, and *Actinobacteria*. These phyla are involved in the production of bile salt hydrolases and alter the structure of bile acids (Jia et al., 2021). Therefore, the seasonal variation of bacterial abundance may be attributed to the differences in hormone entrainment caused by the intensity and duration of seasonal rotation light.

The metabolic rate of the animal organism fluctuates with the turnover of seasons (Zhu and Wang, 2015; Nord et al., 2021). Intestinal bacteria are hidden “organs” involved in regulating host metabolism, energy balance and immune response (Muszer et al., 2015; de Vos et al., 2022). The processes of food digestion, absorption, and nutrient conversion consume energy and release heat, which affect the animal’s body temperature and energy balance (Chan and Hsieh, 2022). The thermal effect of this food accounts for 5 to 15 percent of the total energy expenditure (Okla et al., 2017) and does not change with ambient temperature (DeRuisseau et al., 2004). The ambient temperatures we recorded suggest that growing rabbits may be subjected to heat and cold stress at midday in summer and at night in winter, respectively (Supplementary Figure 8). Compared to spring, the abundance of potentially harmful bacteria increases in summer and winter, the abundance of short-chain fatty acid-producing bacteria decreases, the cecal barrier weakened, and the cecal immune response process activated with greater intensity. The immune response depends on energy availability (Wolowczuk et al., 2008), and changing the timing of feeding under conditions of diurnal ambient temperature fluctuations profoundly interferes with the dynamic processes regulating the balance of energy intake and consumption. Feeding growing rabbits during the hotter daylight days of summer exacerbates the heat stress response by overlapping the food-induced heat production with the thermal environmental periods of the day. Feeding growing rabbits on colder winter nights ensures that the increased energy expenditure due to increased cold-induced thermogenesis is rapidly compensated by caloric intake (Louis-Sylvestre, 1987).

Compared with NRF, DF violates the activity rhythm of growing rabbits, which may disrupt cecal peristaltic rhythm, accumulate ROS, and destroy bacterial balance. It also partially eliminated the regulation of intestinal liver circulation by high concentration of melatonin in dietary stage. Feeding during the day destroys the balance of intestinal microflora, reduces the bacteria that oscillate in circadian rhythm, reduces the characteristics of circadian metabolism, and weakens the function of bacteria to assist host digestion and absorption of nutrients. In particular, daytime feeding in summer causes heat increment effects of food to be distributed during hotter daytime hours, exacerbating the heat stress response (Guo et al., 2021). Daytime feeding keeps the increased heat of the food away from the colder winter nights and does not alleviate cold stress. Therefore, daytime feeding has an impact on the circadian rhythm and diurnal oscillation of cecal bacteria in growing rabbits. This feeding regimen may disrupt digestive coordination and immune response between the host and cecal bacteria due to energy distribution imbalances in high and low temperature environments, exacerbating cold and heat stress responses, thereby increasing the risk of diarrhea.

## 5 Conclusion

In this study, we systematically and comprehensively analyzed the effects of season and feeding time on the composition of rabbit cecum bacteria. This study found a significant correlation between cecum bacteria and seasonal ambient temperature. It also showed that nighttime feeding significantly decreased the abundance of the harmful bacteria in summer and winter, increased the number of cecum bacteria genera with diurnal oscillations, and enhanced the circadian rhythm of bacterial metabolic activity. In general, different seasons and feeding time interacted to influence the composition and circadian rhythms of cecum bacteria. We suggest that synchronization of nutrient signals altered by foraging activity with the light-dark cycle is particularly important for the dynamic balance of the cecum bacteria. Seasonal and feeding time “mismatches” can lead to imbalances in gut bacteria, disrupting their balance and natural rhythms, which may increase the risk of diarrhea.

## Data availability statement

The datasets presented in this study can be found in online repositories. The names of the repository/repositories and accession number(s) can be accessed at https://www.ncbi.nlm.nih.gov/sra, registration number PRJNA632844.

## Ethics statement

The animal studies were approved by the China Agricultural University Laboratory Animal Welfare and Animal Experimental Ethical Committee. The studies were conducted in accordance with the local legislation and institutional requirements. Written informed consent was obtained from the owners for the participation of their animals in this study.

## Author contributions

SH: Methodology, Visualization, Formal analysis, Writing – original draft, Writing – review and editing. K-HZ: Methodology, Investigation, Data curation, Writing – review and editing. Q-YJ: Methodology, Formal analysis, Visualization, Writing – review and editing. Q-JW: Conceptualization, Methodology, Formal analysis, Visualization, Writing – review and editing. JH: Data curation, Investigation, Writing – review and editing. J-JL: Data curation, Investigation, Writing – review and editing. YG: Investigation, Conceptualization, Writing – review and editing. PL: Investigation, Conceptualization, Writing – review and editing. Z-YL: Investigation, Conceptualization, Writing – review and editing. DL: Investigation, Data curation, Writing – review and editing. S-XG: Investigation, Data curation, Writing – review and editing. QL: Investigation, Data curation, Writing – review and editing. M-YL: Investigation, Conceptualization, Data curation, Writing – review and editing. ML: Investigation, Conceptualization, Data curation, Writing – review and editing. Z-HW: Conceptualization, Funding acquisition, Supervision, Writing – review and editing.

## Footnotes

1
http://www.biocloud.net

2
https://cran.r-project.org/web/packages/psych/index.html
