# Supplementary material for: The effects of ambient temperature and feeding regimens on cecum bacteria composition and circadian rhythm in growing rabbits
Source: Front Microbiol. 2024 Feb 27;15:1344992. doi: 10.3389/fmicb.2024.1344992 (PMC10927733; doi:10.3389/fmicb.2024.1344992)
Supplement: Supplementary file 1 [file Data_Sheet_1.zip › Presentation 1.pdf]

Supplementary Material Presentation

Supplementary Figure S1. Rarefaction curves based on Shannon index at the ASV level of growing rabbits.
Supplementary Figure S2. Analysis of cecal bacteria α- and β- diversity in growing rabbits in three seasons. (A) ACE index analysis of cecum bacteria. (B) Nonmetric multidimensional scaling (NMDS) analysis of cecum bacteria. (C) PCoA analysis of cecum bacteria.
Supplementary Figure S3. Composition of intestinal bacterial phylum levels in rabbits grown under different feeding regimens.
Supplementary Figure S4. Cecal rhythmic ASVs in DF and NRF growing rabbits in spring.
Supplementary Figure S5. Changes in the relative abundance of cecum bacteria across feeding regimens and seasons. (A) Simple effect analysis of the season × feeding time interaction term for the relative abundance of *Ruminococcus_1*. (B) Other bacteria have the interaction effect of season and feeding time. The "*" on the column indicates the variability of different feeding regimens under the same season; "*" between the columns indicates the variability between different seasons under the same feeding regimen; "*" represents $P < 0.05$ and "**" represents $P < 0.01$.
Supplementary Figure S6. Rank-sum test of cecum bacteria at the genus levels in three seasons.
Supplementary Figure S7. Temperature changes in growing rabbit shed in summer, spring and winter. (A), The temperature changes during feeding in summer, spring and winter. (B) 24h temperature variation in summer, spring and winter rabbit sheds, mean ± standard deviation; Grey background means night.
Supplementary Figure S8. PICRUSt predicted the diurnal metabolic features of cecum bacteria for different feeding regimens in (A) winter, in (B) spring. The stacked histograms show the values of the metabolic potential of the bacteria predicted by PICRUSt for the individual time points and for the set composed of the remaining time points of the same day.
