# Supplementary figures and images for: The effects of ambient temperature and feeding regimens on cecum bacteria composition and circadian rhythm in growing rabbits
Source: Front Microbiol. 2024 Feb 27;15:1344992. doi: 10.3389/fmicb.2024.1344992 (PMC10927733; doi:10.3389/fmicb.2024.1344992)

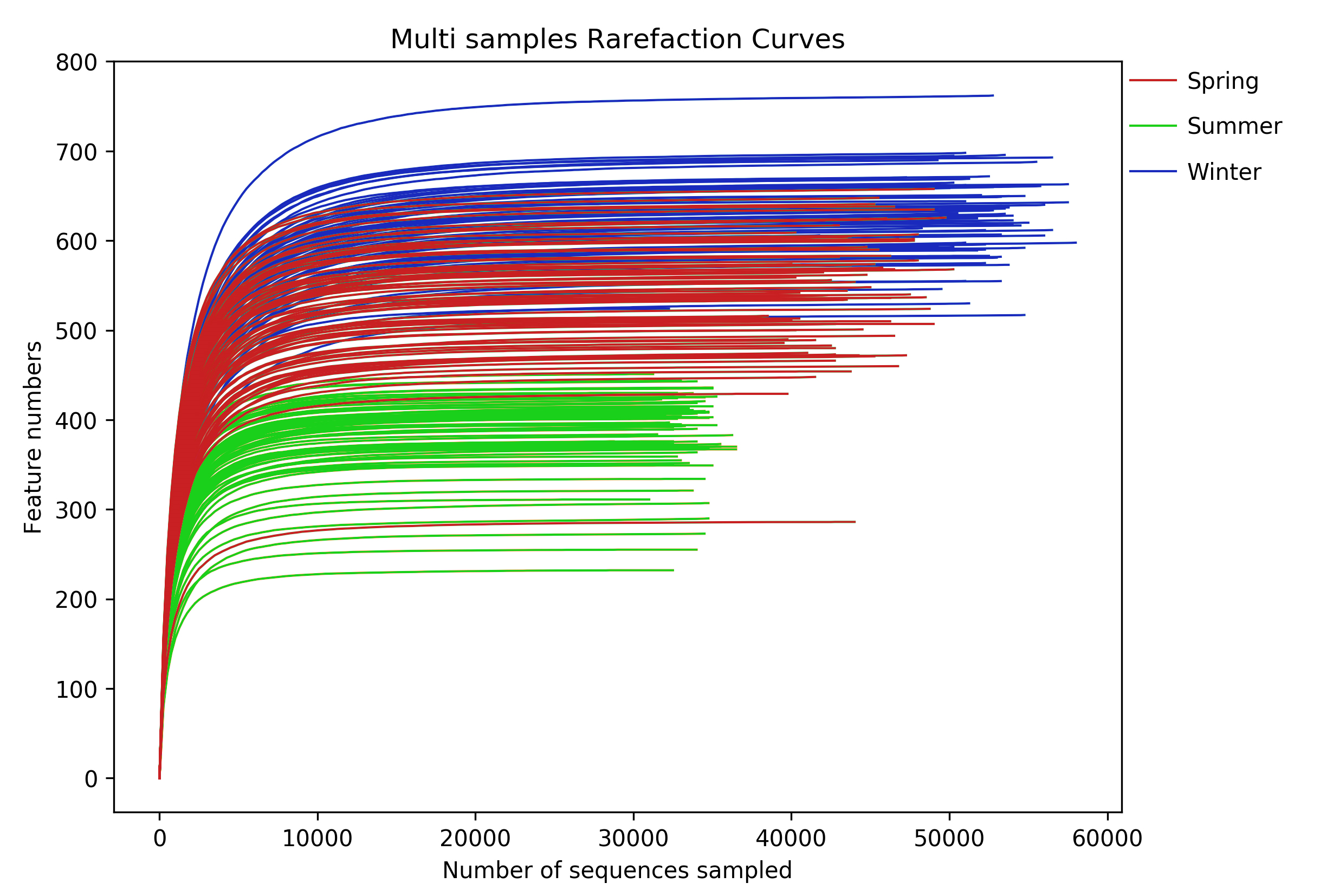

Supplement: Supplementary file 1 [file Data_Sheet_1.zip › Supplementary Figure S1. Rarefaction curves based on Shannon index at the ASV level of growing rabbits.tif]

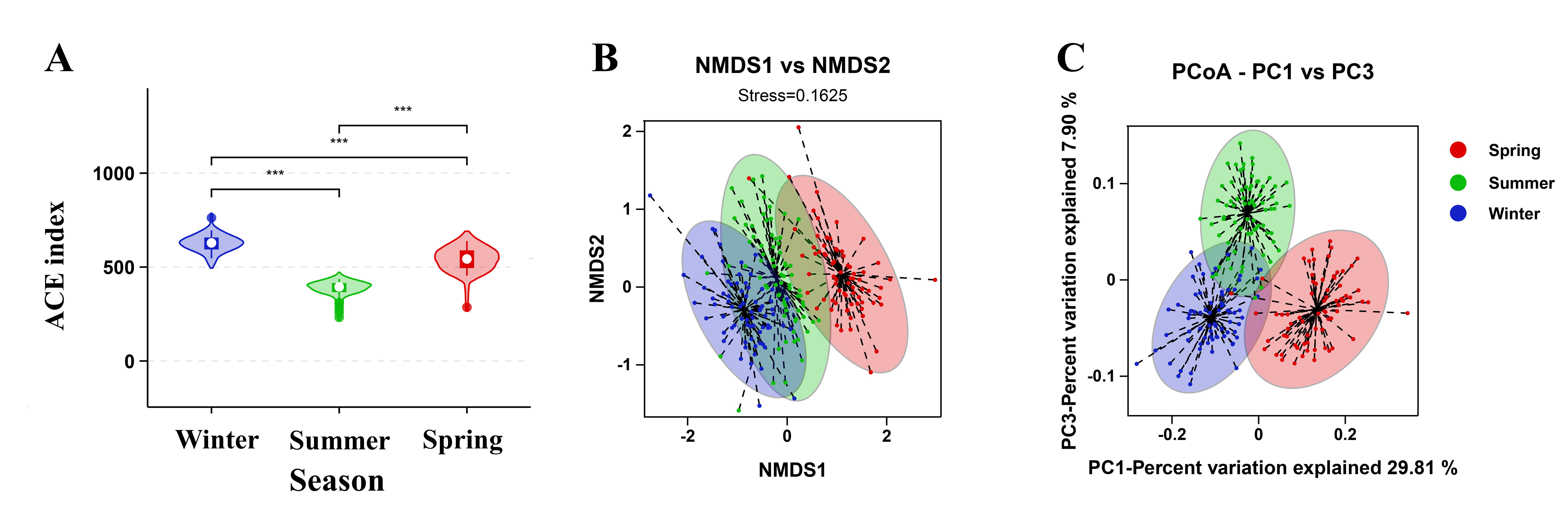

Supplement: Supplementary file 1 [file Data_Sheet_1.zip › Supplementary Figure S2. Analysis of cecal bacteria x and x diversity in growing rabbits in three seasons..png]

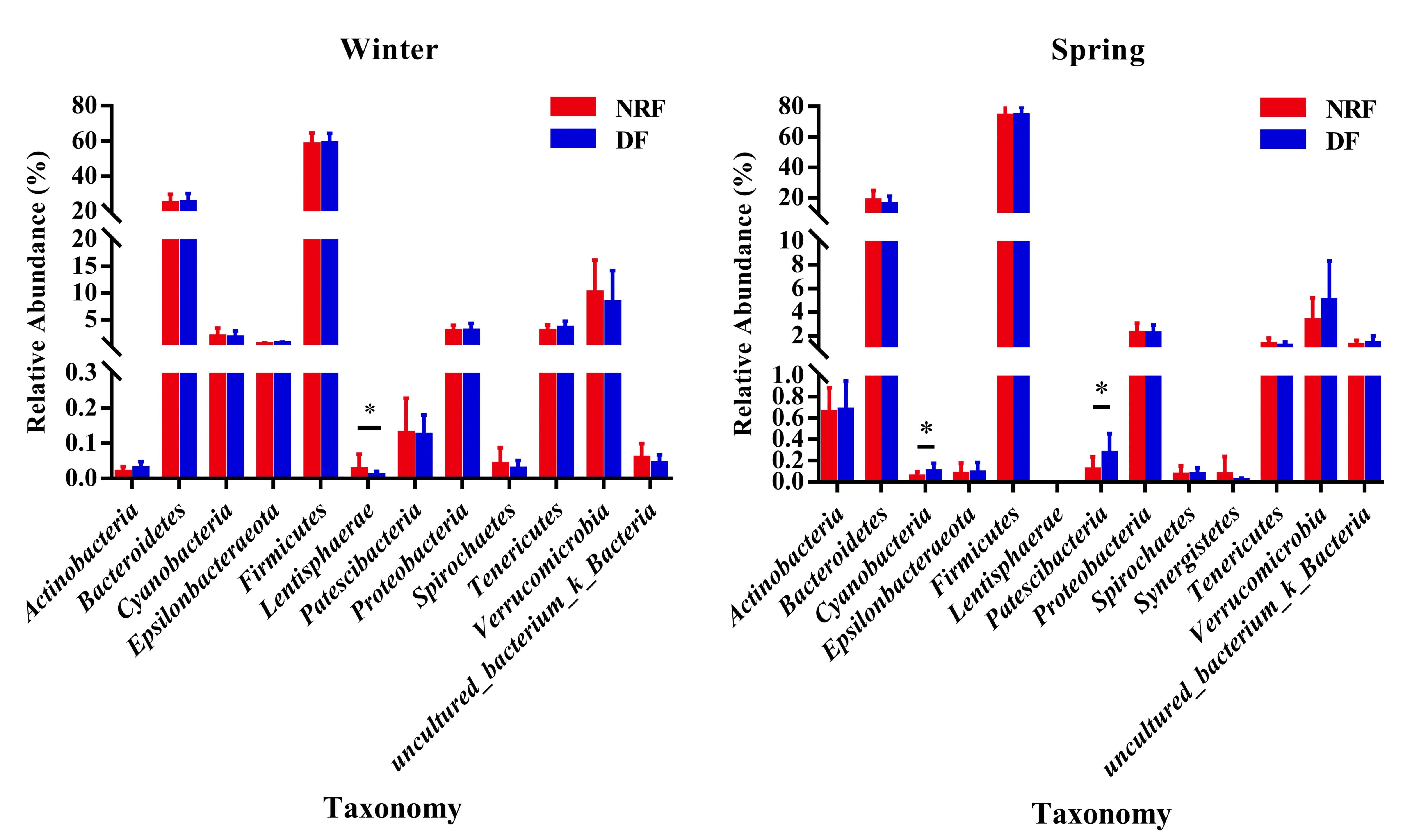

Supplement: Supplementary file 1 [file Data_Sheet_1.zip › Supplementary Figure S3. Composition of intestinal bacterial phylum levels in rabbits grown under.tif]

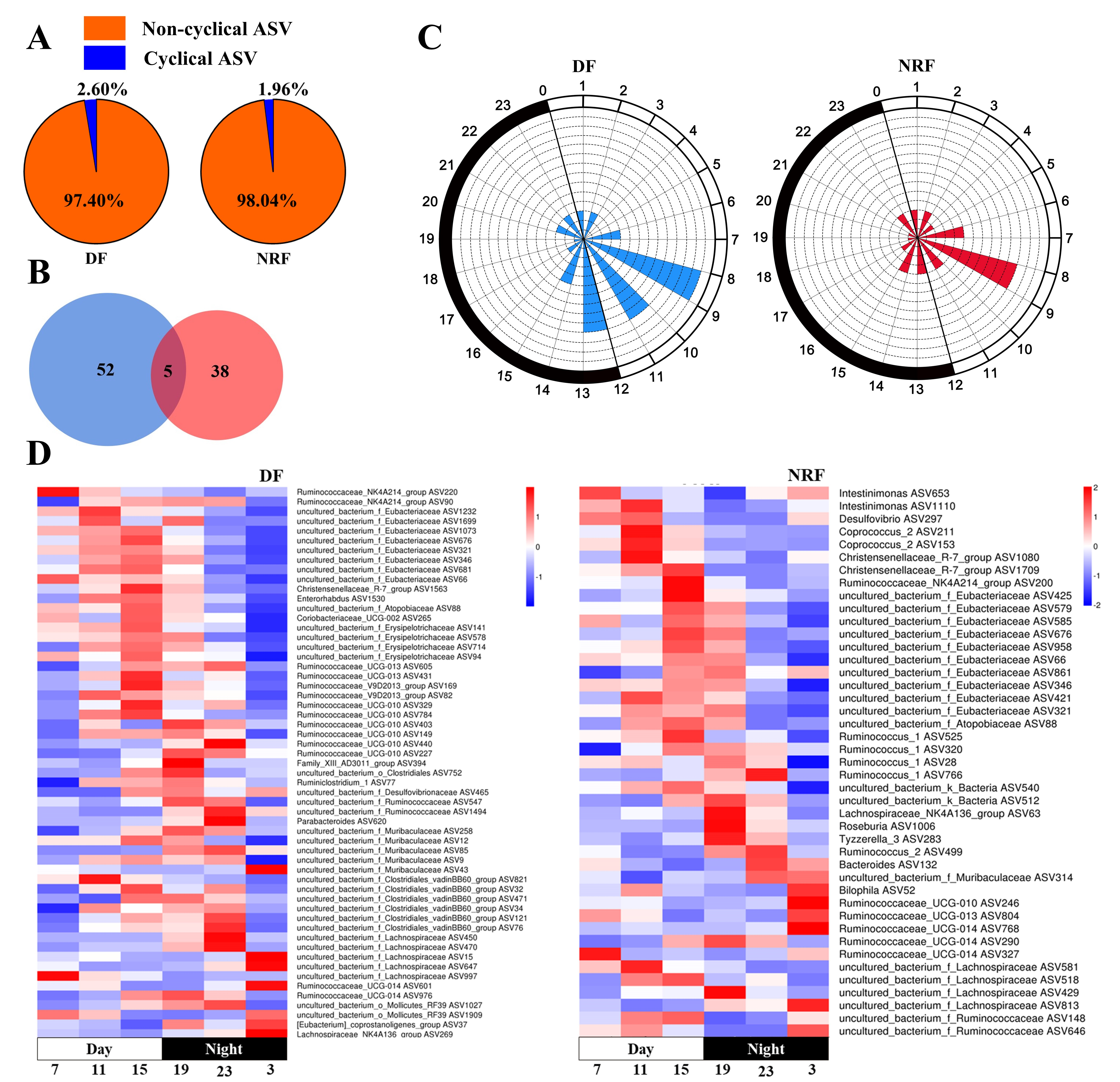

Supplement: Supplementary file 1 [file Data_Sheet_1.zip › Supplementary Figure S4. Cecal rhythmic ASVs in DF and NRF growing rabbits in spring.tif]

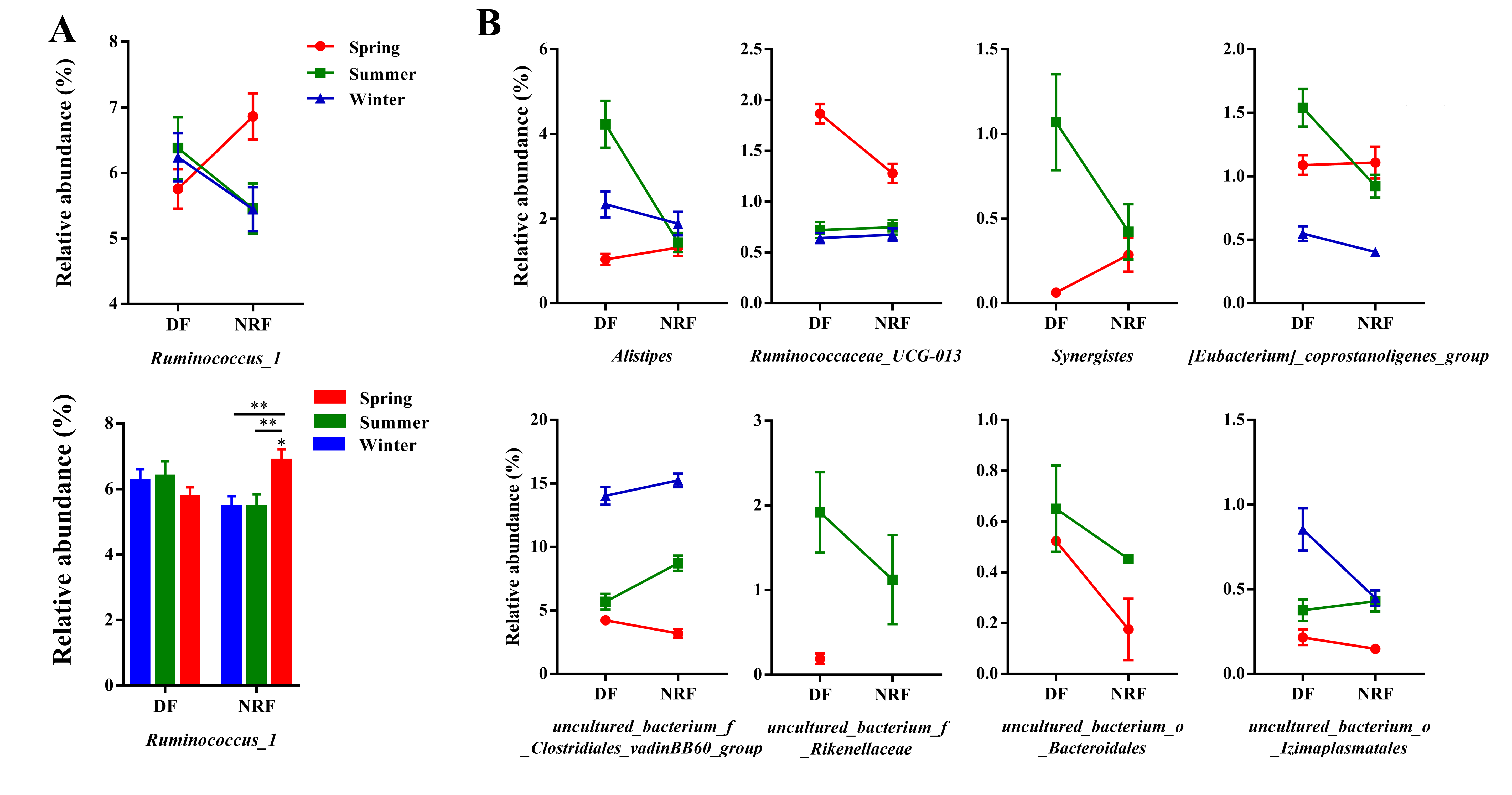

Supplement: Supplementary file 1 [file Data_Sheet_1.zip › Supplementary Figure S5. Changes in the abundance of cecum bacteria across feeding regimens and seasons.png]

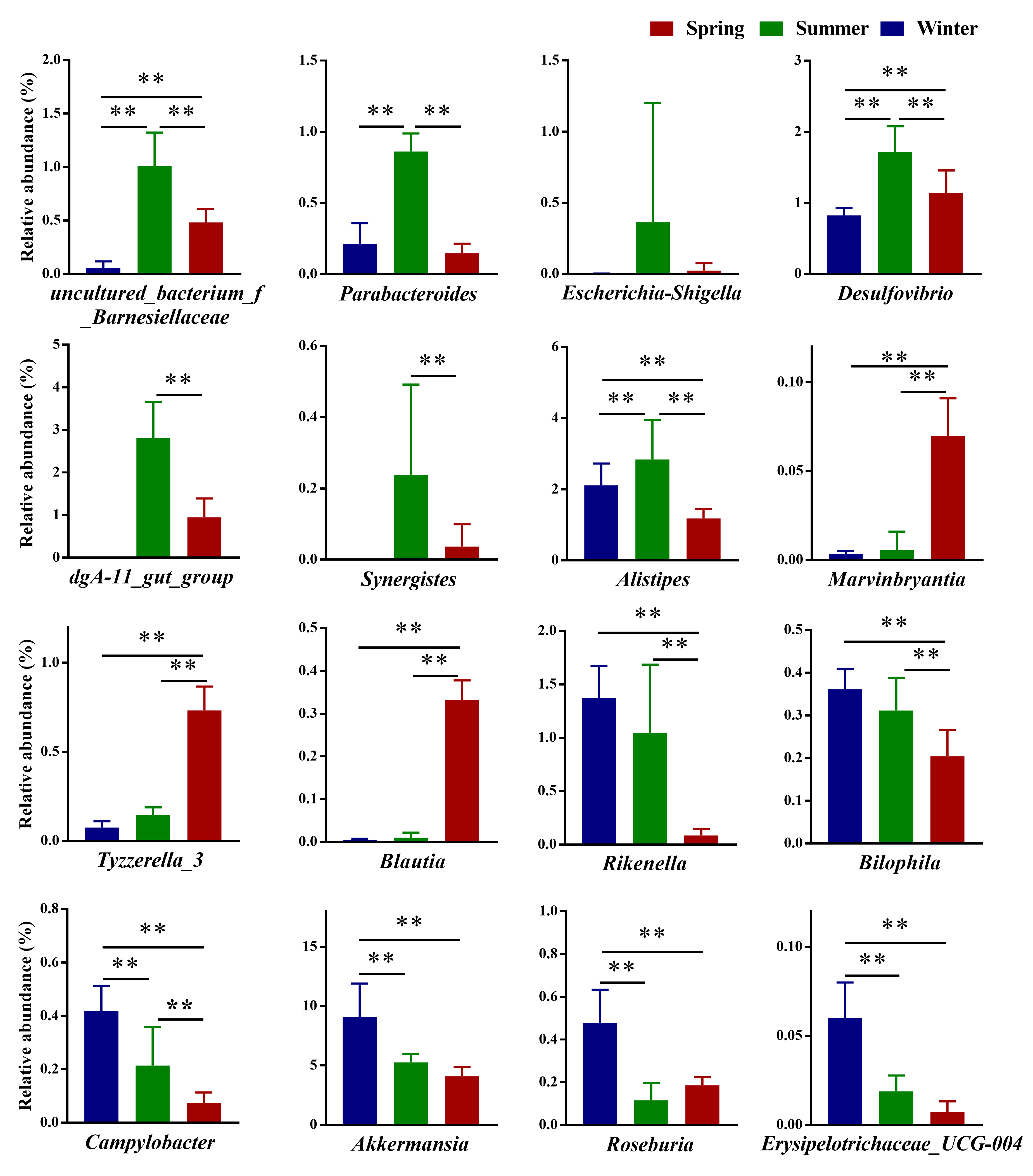

Supplement: Supplementary file 1 [file Data_Sheet_1.zip › Supplementary Figure S6. Rank-sum test of cecum bacteria at the genus levels in three seasons.tif]

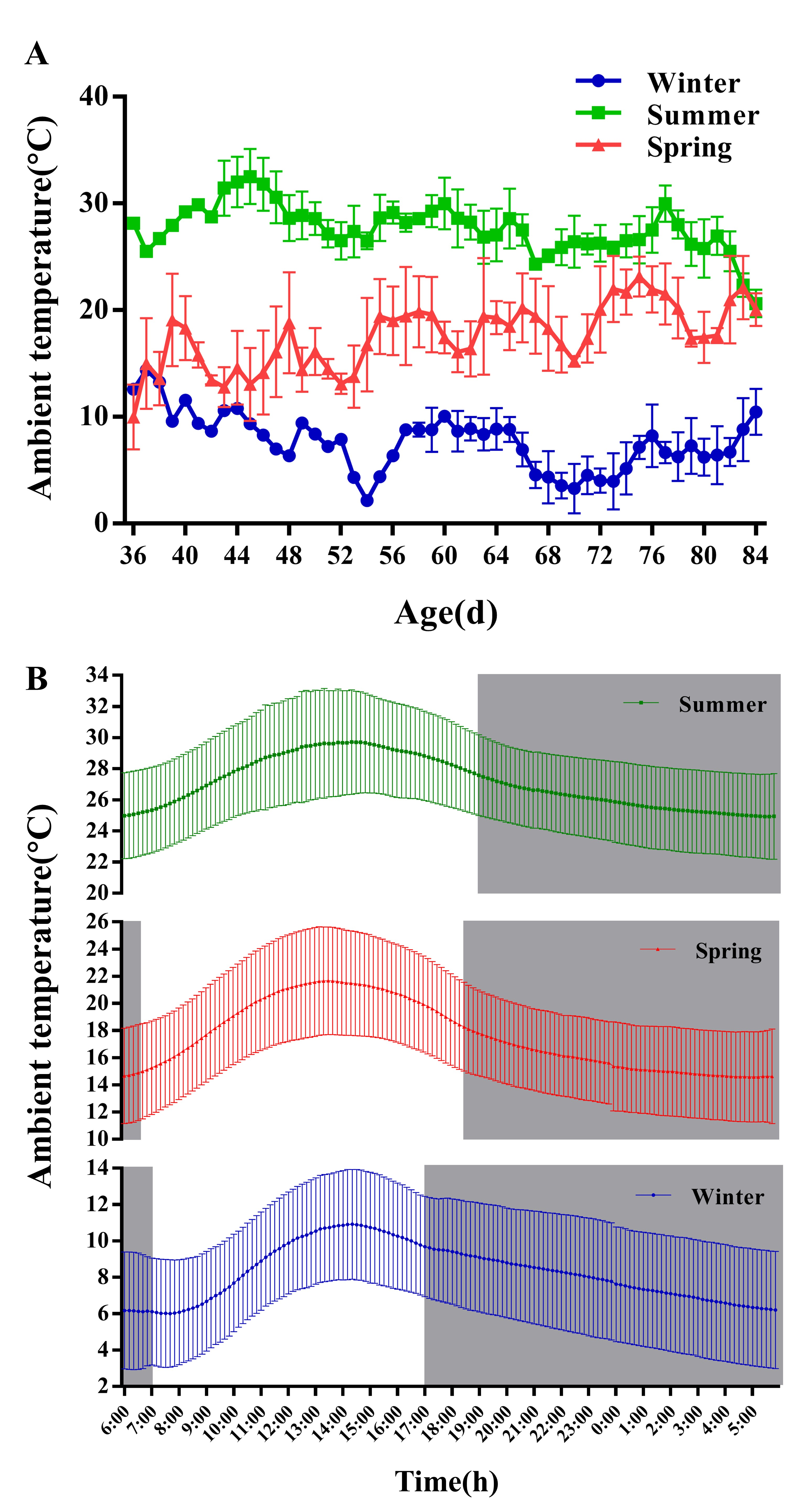

Supplement: Supplementary file 1 [file Data_Sheet_1.zip › Supplementary Figure S7. Temperature changes in growing rabbit shed in summer, spring and winter.tif]

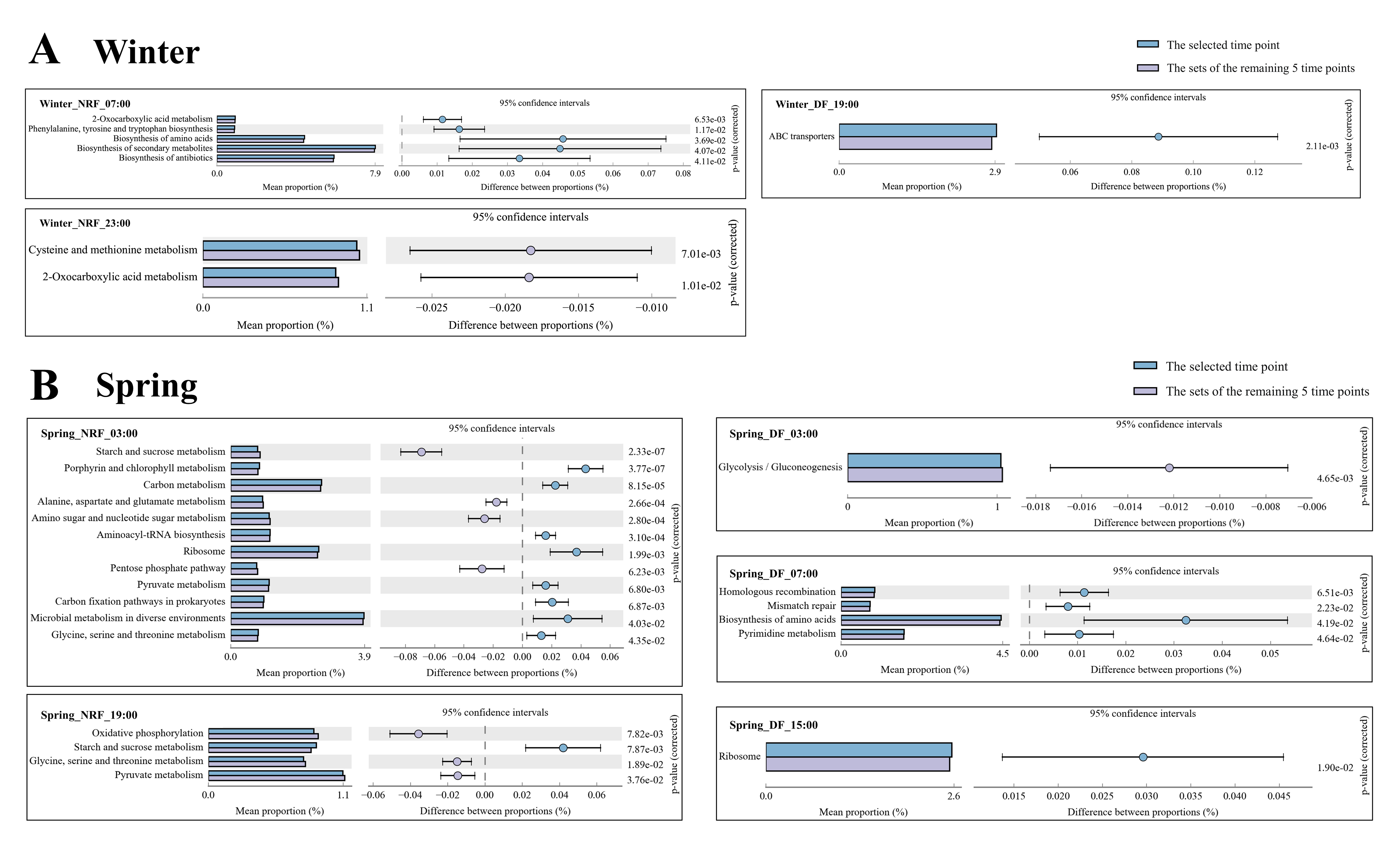

Supplement: Supplementary file 1 [file Data_Sheet_1.zip › Supplementary Figure S8. PICRUSt predicted the diurnal metabolic features of cecum bacteria .png]
